# Tranexamic acid administration and pulmonary embolism in combat casualties with orthopaedic injuries

**DOI:** 10.1097/OI9.0000000000000143

**Published:** 2021-10-19

**Authors:** Benjamin W. Hoyt, Michael D. Baird, Seth Schobel, Henry Robertson, Ravi Sanka, Benjamin K. Potter, Matthew Bradley, John Oh, Eric A. Elster

**Affiliations:** a USU-WRNMMC Department of Surgery; b Surgical Critical Care Initiative (SC2i), Uniformed Services University of the Health Sciences, Bethesda; c Henry Jackson Foundation, Rockville, MD USA; d Naval Medical Research Center, Silver Spring, Maryland.

**Keywords:** amputation, combat-related trauma, pulmonary embolism, tranexamic acid, venous thromboembolism

## Abstract

**Objectives:**

In combat casualty care, tranexamic acid (TXA) is administered as part of initial resuscitation effort; however, conflicting data exist as to whether TXA contributes to increased risk of venous thromboembolism (VTE). The purpose of this study is to determine what factors increase risk of pulmonary embolism after combat-related orthopaedic trauma and whether administration of TXA is an independent risk factor for major thromboembolic events.

**Setting:**

United States Military Trauma Centers.

**Patients:**

Combat casualties with orthopaedic injuries treated at any US military trauma center for traumatic injuries sustained from January 2011 through December 2015. In total, 493 patients were identified.

**Intervention:**

None.

**Main Outcome Measures:**

Occurrence of major thromboembolic events, defined as segmental or greater pulmonary embolism or thromboembolism-associated pulseless electrical activity.

**Results:**

Regression analysis revealed TXA administration, traumatic amputation, acute kidney failure, and hypertension to be associated with the development of a major thromboembolic event for all models. Injury characteristics independently associated with risk of major VTE were Injury Severity Score 23 or greater, traumatic amputation, and vertebral fracture. The best performing model utilized had an area under curve  = 0.84, a sensitivity=0.72, and a specificity=0.84.

**Conclusions:**

TXA is an independent risk factor for major VTE after combat-related Orthopaedic injury. Injury factors including severe trauma, major extremity amputation, and vertebral fracture should prompt suspicion for increased risk of major thromboembolic events and increased threshold for TXA use if no major hemorrhage is present.

**Level of evidence:**

III, Prognostic Study

## 1 Introduction

Orthopaedic extremity injuries, including traumatic amputations, represent over 80% of combat injuries sustained in the recent military conflicts in Iraq and Afghanistan, with the majority of fractures being open and at high risk for complications and poor outcomes. In service members injured in combat, tranexamic acid (TXA) is frequently administered following severe injury as part of resuscitation efforts. Patients who sustain acute hemorrhagic trauma are at an increased risk of coagulopathy; TXA inhibits fibrinolysis, which acts to stabilize clots and limit blood loss.^[1]^

TXA use provided a mortality benefit in both the civilian and military trauma populations in the Clinical Randomization of an Antifibrinolytic in Significant Haemorrage (CRASH)-2 trial and Military Application of Tranexamic Acid in Trauma Emergency Resuscitation (MATTERs) trials, respectively.^[2,3]^ The CRASH-2 study was a randomized controlled trial of over 20,000 patients who received a 1 g dose of TXA over 10 minutes followed by a 1 g infusion over the next 8 hours, lowering all cause mortality as well as the risk of death due to hemorrhage.^[2,4]^ The MATTERs study was a retrospective study of 896 combat-injured patients, 293 of whom received a 1 g bolus of TXA at the discretion of the surgeon or anesthesiologist or when hyperfibrinolysis was suspected, that found reduced mortality and coagulopathy in the group that received TXA despite higher injury severity.^[3]^ Following CRASH-2, TXA was added to the Tactical Combat Casualty Care guidelines and the Joint Trauma Service clinical practice guidelines for damage control resuscitation and prolonged field care as a method for decreasing massive transfusion and improving mortality.^[5–7]^ In orthopaedic surgery, TXA has been used to safely decrease perioperative bleeding and the need for transfusion for over 4 decades.^[8]^

While initial trauma studies did not show a relationship between TXA administration and venous thromboembolism (VTE), concerns have been raised in subsequent studies.^[9,10]^ These studies have focused largely on all thromboembolic events, including both deep venous thrombosis (DVT) and pulmonary embolism (PE). However, these outcome measures may be too broad to guide clinical decision-making. PE is a leading cause of in-hospital mortality and readmission for trauma patients,^[11]^ but the size and location matter. Isolated subsegmental PE are over-diagnosed, are not associated with increased mortality, and their treatment with thrombolytics is associated with increased iatrogenic hemorrhagic complications without improving mortality outcomes.^[12–17]^ Recent CHEST guidelines recommend monitoring over anticoagulation in the setting of these small emboli.^[18]^ While some studies have reported worse outcomes for patients with concomitant DVT and PE,^[19,20]^ isolated DVT carries limited clinical significance for decision-making in the trauma care setting. Lower extremity DVT, particularly distal DVTs, may have limited systemic impact, with some evidence suggesting they do not correlate with increased frequency of PE or overall mortality after trauma.^[21–25]^ It may therefore be detrimental to evaluate the safety and complications of a potentially life-saving intervention using questionably relevant clinical endpoints.

The purpose of this study was therefore to determine if TXA administration and other patient or treatment-related factors alter the risk for, and can be predictive of, *major* thromboembolic events in patients with severe traumatic orthopaedic injuries. We defined a major VTE as PE and/or VTE-associated pulseless electrical activity, excluding isolated subsegmental PE and peripheral DVT given their low clinical risk. We hypothesized that TXA administration, trauma severity, and presence of a DVT would be associated with increased likelihood of major VTE event.

## 2 Methods

### 2.1 Study design and patient selection

After approval by our hospital's institutional review board, we performed a query of the Department of Defense Trauma Registry for all combat-injured patients necessitating medical evacuation from forward surgical care in Iraq or Afghanistan to a military trauma center within the United States. This research was approved by our internal review board for research ethics and was conducted in accordance with the Declaration of the World Medical Association. Time periods considered were January 2011 to December 2015. Patients were included if they were United States military, non-United States military, or civilian contractors, and trauma included at least 1 orthopaedic injury. Patients were excluded for care terminating at initial level of treatment or outside the continental US. Three patients were excluded for death prior to arrival to the United States. In total, 493 consecutive patients were included for analysis.

### 2.2 Data collection

Demographic and injury characteristics were collected for all patients. Data points collected were specifics of orthopaedic injuries to include extremity fractures, extremity neurovascular injuries, and traumatic amputations. Therapeutic procedures including administration of blood products, antithrombolytic agents, and diagnostic procedures (e.g., lower extremity ultrasound) were also documented. Additionally, clinical events including deep venous thrombosis, infection, kidney failure, and rhabdomyolysis were collected. Our primary outcome measure was the development of a major thromboembolic event, which we defined as any pulmonary embolism diagnosis confirmed as more proximal than subsegmental by computed tomography or the diagnosis of a thromboembolism-associated pulseless electrical activity event based on clinical documentation by the care team.

Within injury types, hierarchical variables were created to create a greater clinical relevance and to ensure that redundancies and subtle differences in medical International Classification of Diseases-9 billing codes did not impact data analysis. Within orthopaedic injuries, more specific derived variables included: vertebral fracture, cord injury, pelvic fracture, shoulder fracture, humerus fracture, forearm fracture, hand/finger fracture, femoral fracture, lower leg fracture, and toe/foot fracture. Larger subgroups were created and encompassed the aforementioned variables to create the categories of open fracture, upper extremity fracture (open and closed), and lower extremity fracture (open and closed). Other specific nonosseous variables include amputation, upper extremity vascular injury, lower extremity vascular injury, upper extremity nerve injury, and lower extremity nerve injury. Larger nonosseous subgroups were both vascular injury and nerve injury. Finally, the variables of upper extremity injury, lower extremity injury, and spinal column injury were representative of both bony and non-bony orthopaedic injuries.

### 2.3 Statistical analysis

To avoid the bias of complete case analysis, multiple imputation by random forest was done to fill missing values in a small number of predictor variables. Univariate analyses were applied to assess relationships between major thromboembolic event and demographic, injury, clinical, and treatment characteristics. Following univariate analysis, logistic least absolute shrinkage and selection operator (LASSO) regression with and without forced inclusion of Injury Severity Score (ISS) and age was used to identify independent variables associated with the outcome. Candidate variables for multivariate analysis were identified based on significance or near-significance (*P* < .15) on univariate analysis; 126 variables or variable groupings met this criteria. The model utilizing forced inclusion of ISS was performed to ensure the covariates identified by LASSO regression were independent of increased trauma severity, an established confounder for VTE after injury.^[26,27]^ A regression was also performed exclusively for injury characteristics. Hundred-fold cross-validations were done for all models to obtain robust estimates of fit statistics. The best performing models from these analyses were selected on the basis of a variety of performance statistics including area under curve, sensitivity, and specificity.

## 3 Results

Four hundred ninety-three patients were medically evacuated for injuries including orthopaedic injuries during the study period. Forty-six patients (9.3%) were diagnosed with major thromboembolic events at military treatment facilities in the continental US or en route to the US. In the same cohort, a total of 56 patients (11.4%) were diagnosed with DVT and 62 were diagnosed with PE (12.6%) based on CT angiography—of these 9 patients were diagnosed with both DVT and PE for a total 109 patients (22.1%) with any diagnosis of VTE. Patients were predominately male (99.4%) and sustained injuries from a blast/explosive mechanism in 80.1% of cases with mean ISS 21.5 ± 12 (Table 1). Forty percent of injuries included at least 1 traumatic extremity amputation. 35.7% of patients received intravenous TXA as part of their early treatment course.

**Table 1:** Baseline demographics and clinical characteristics

Mean patient age (years)	24.6 ± 5.1
Male sex (%)	490 (99.4)
Tobacco use (%)	188 (38.1)
Blast mechanism (%)	395 (80.1)
Mean ISS	21.5 ± 12
Open wound (%)	450 (91.3)
Lower extremity fracture present (%)	398 (80.7)
Upper extremity fracture present (%)	213 (43.2)
Traumatic amputation (%)	197 (40)
Time before arrival to definitive care (days)	2 ± 0.3
Mean time on ventilator (days)	4.5 ± 6.1
Mean time hospitalized (days)	26.1 (27.5)
TXA administered	176 (35.7)
DVT diagnosed	56 (11.4)
Any PE diagnosed	62 (12.6)
Major thromboembolic event (%)	46 (9.3)

**Figure 1:**
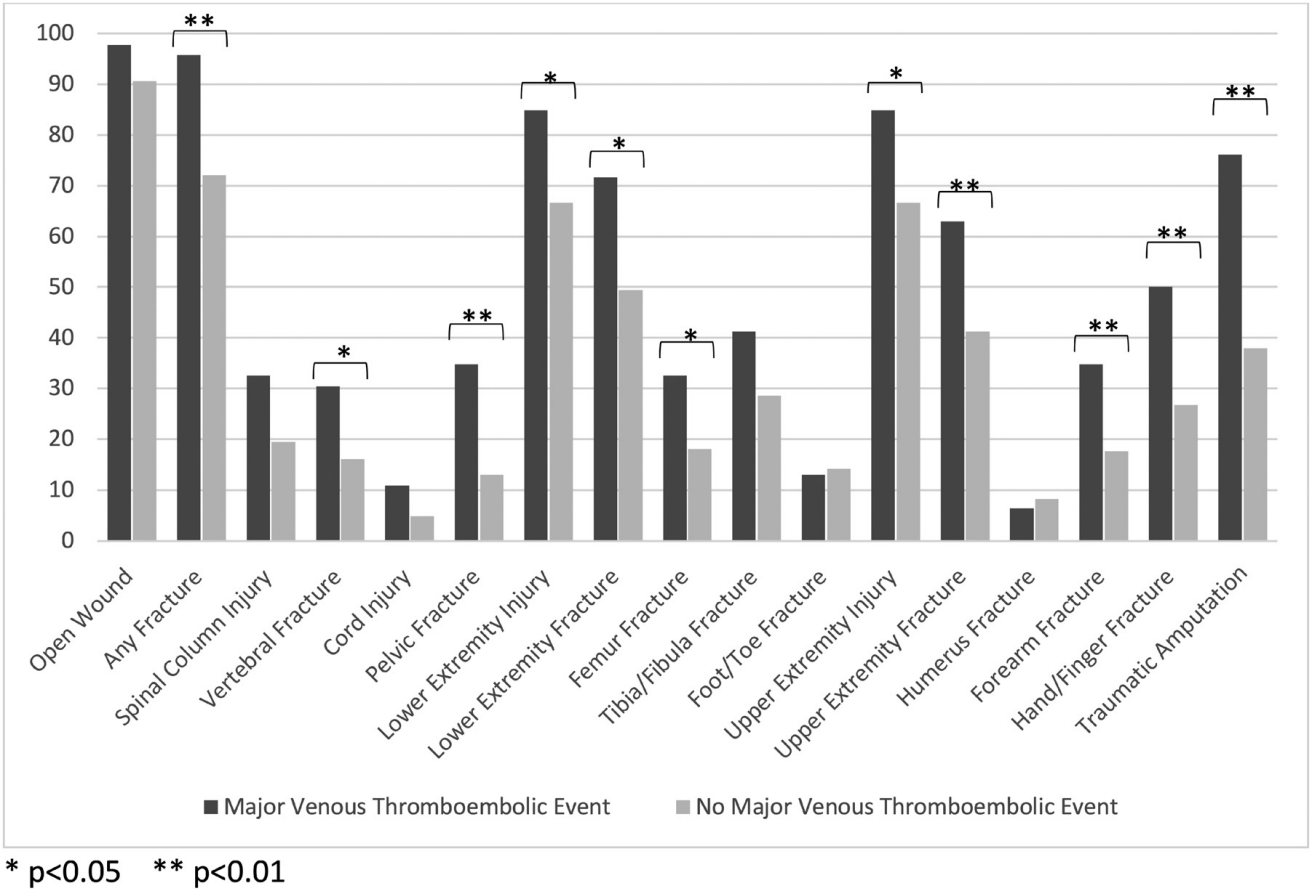
Incidence of orthopaedic injuries in the setting of major VTE event diagnosis.

Out of a total of 126 candidate predictor variables, 114 (90.5%) had complete data; the remaining 8 variables had missingness ranging from 0.1% to 36.3%. The missing values were filled in through multiple imputations by random forest. None of the imputed variables emerged as strong predictors in the LASSO models. The outcome of major thromboembolic events had no missing data; injury characteristics associated with this outcome in univariate analysis included composite ISS, initial base deficit, higher initial international normalized ratio, blast injury mechanism, and presence of fracture or traumatic amputation (Table 2, Fig. 1). Notably, presence of a diagnosed DVT and a history of prior DVT/PE was not associated with major thromboembolic events (*P* = .15 and *P* = .45, respectively).

**Table 2:** Univariate regression analysis

Factor	Major VTE (n = 46)	No major VTE (n = 447)	*P*
Patient			
Age (years)	24.6	24.6	.94
Male sex (%)	46 (100)	444 (99.3)	1
Tobacco use (%)	16 (34.8)	172 (38.5)	.74
History of DVT/PE (%)	1 (2.2)	1 (0.2)	.45
Injury/initial care			
Composite ISS	30.2	20.6	<.001^∗^
Mechanism: explosives (%)	43 (93.5)	352 (78.7)	.029^∗^
GCS on arrival to initial care	10.1	12.5	.004^∗^
Initial base deficit	7.4	4.5	.002^∗^
Initial INR	1.4	1.3	.004^∗^
Mean blood Products first 24 hours (units)	33.4	18.5	.001^∗^
TXA administered (%)	34 (73.9)	142 (31.8)	<.001^∗^
Factor VII administered (%)	0 (0)	11 (2.5)	.58
FFP administered (%)	4 (8.7)	10 (2.2)	.041^∗^
Orthopaedic injuries			
Open wound (%)	45 (97.8)	405 (90.6)	.17
Any fracture (%)	44 (95.7)	322 (72)	<.001^∗^
Spinal column Injury (%)	15 (32.6)	87 (19.5)	.06
Vertebral fracture (%)	14 (30.4)	72 (16.1)	.026^∗^
Cord injury (%)	5 (10.9)	22 (4.9)	.18
Pelvic fracture (%)	16 (34.8)	58 (13)	<.001^∗^
Lower extremity injury (%)	39 (84.8)	298 (66.7)	.019^∗^
Lower extremity vascular injury (%)	6 (13)	64 (14.3)	.99
Lower extremity nerve injury (%)	4 (8.7)	22 (4.9)	.46
Lower extremity fracture (%)	33 (71.7)	221 (49.4)	.006^∗^
Femur fracture (%)	15 (32.6)	81 (18.1)	.030^∗^
Tibia/fibula fracture (%)	19 (41.3)	128 (28.6)	.11
Foot/toe fracture (%)	6 (13)	64 (14.3)	.99
Upper extremity injury (%)	39 (84.8)	298 (66.7)	.019^∗^
Upper extremity vascular injury (%)	4 (8.7)	47 (10.5)	.9
Upper extremity nerve injury (%)	4 (8.7)	49 (11)	.82
Upper extremity fracture (%)	29 (63)	184 (41.2)	.007^∗^
Humerus fracture (%)	3 (6.5)	37 (8.3)	.9
Forearm fracture (%)	16 (34.8)	79 (17.7)	.009^∗^
Hand/finger fracture (%)	23 (50)	120 (26.8)	.002^∗^
Traumatic amputation (%)	35 (76.1)	170 (38)	<.001^∗^
Additional diagnoses			
Rhabdomyolysis (%)	12 (26.1)	55 (12.3)	.018^∗^
Acute kidney injury (%)	5 (10.9)	4 (0.9)	<.001^∗^
Hypertension (%)	4 (8.7)	5 (1.1)	.002^∗^
Cardiac pathology (%)	4 (8.7)	5 (1.1)	.002^∗^
Diagnosed DVT (%)	8 (17.4)	42 (9.4)	.15
VTE consequences			
Respiratory failure (%)	4 (8.7)	6 (1.3)	.005^∗^
Time in hospital (days)	41.2	24.5	<.001^∗^
HIT (%)	2 (4.3)	0 (0)	.001^∗^

∗ *P* < .05

HIT = heparin-induced thrombocytopenia; INR = international normalized ratio.

Administration of TXA was associated with a higher rate of the primary outcome of major VTE (73.9% vs 31.8%, *P* < .001). Major thromboembolic event was associated with adverse outcomes including heparin-induced thrombocytopenia (4.3% vs 0%, *P* = .001) and prolonged hospital stay (41.2 vs 24.5 days, *P* < .001). However, these outcomes were also associated with other factors in the model including ISS and traumatic amputation (*P* < .001).

Analysis using multivariate regression revealed several variables—TXA administration, traumatic amputation, acute kidney failure, and a medical history including hypertension—to be most predictive for development or presence of a major thromboembolic event in both models (Tables 3 and 4). The logistic regressions found a low baseline risk of VTE and several positive risk factors. The best performing models included TXA administration and sedation at the time of transfer to definitive care and increased time on ventilator as predictive factors. Injury characteristics most predictive of VTE included traumatic amputation, ISS 23 or greater, and vertebral fracture (Table 5). The best performing model utilized had an area under curve of 0.84 (0.83–0.84), a sensitivity of 0.69 (0.67–0.71), and a specificity of 0.84 (0.82–0.86). The models performed similarly whether or not ISS was included; various risk factors were positively correlated with one another and interchangeable in a data set of this size.

**Table 3:** Results of multivariate regression model with forced inclusion of ISS

Rank	Factor	Log odds	Standard error	*P* value
NA	Intercept	–4.97	0.55	<.001
Forced	Composite ISS	0.04	0.01	.013^∗^
1	TXA administered	1.02	0.38	.008^∗^
2	Diagnosed anemia	0.78	0.34	.023^∗^
3	Infection during hospitalization	0.79	0.35	.024^∗^
4	Sedated at transfer	0.98	0.47	.037^∗^

∗ *P* < .05 AUC 0.82 (0.79–0.81). Sens 0.83 (0.69–0.73). Spec 0.74 (0.84–0.88).

**Table 4:** Results of multivariate regression model without ISS

Rank	Variable	Log odds	Standard error	*P* value
NA	(Intercept)	–5.47	1.03	<.001^∗^
Forced	Age	0.04	0.03	.23
1	TXA administered	0.95	0.43	.028^∗^
2	Total days ventilator	0.07	0.02	<.001^∗^
3	Traumatic amputation	0.98	0.42	.021^∗^
4	Sedated at transfer	0.92	0.48	.05^∗^

∗ *P* < .05 AUC 0.82 (0.79–0.81). Sens 0.89 (0.81–0.85). Spec 0.64 (0.70–0.74).

**Table 5:** Results of multivariate regression model for injury characteristics

Rank	Factor	Log odds	Standard error	*P* value
NA	Intercept	–4.31	0.23	<.0001^∗^
1	Traumatic amputation	1.33	0.20	<.0001^∗^
2	ISS >=23	1.73	0.22	<.0001^∗^
3	Vertebral fracture	0.83	0.20	<.0001^∗^

∗ *P* < .05

AUC = 0.80 (0.79–0.81); sensitivity = 0.73 (0.73–0.74); specificity = 0.81 (0.80–0.82).

## 4 Discussion

While previous data suggests TXA may reduce hemorrhage-related mortality in appropriately selected patients, the risk of major thromboembolic events remains a concern following orthopaedic polytrauma. Much of the recent doctrine in trauma is due to the CRASH-2 trial.^[2]^ In this trial, patients had lower all-cause and hemorrhage-related mortality. Additionally, no significant differences were reported in VTE events with the administration of TXA, though the authors have acknowledged that VTE events may have been underreported and specifically state that they could not rule out possible risk with TXA administration.^[2,4,28]^ Current guidelines for TXA utilization may be too broad and result in overuse in lower hemorrhage risk military trauma, and TXA use appears to represent an independent risk factor for VTE. Johnston et al^[10]^ called for further research into improved calibration of TXA use for battlefield injuries. Our model, which focuses on orthopaedic injuries, is 1 step toward better calibrating TXA use by better understanding risk factors for clinically important adverse outcomes.

Military-specific injuries are associated with a higher incidence of VTE, as well as an increased ISS, when comparing the MATTERs and CRASH-2 studies.^[2–4]^ The military also has a far higher rate of amputation after trauma than the general population. The CRASH-2 data also predominantly relied on blunt trauma rather than the blast or bullet wounds that are more prevalent in our military study population. Gillern et al^[24]^ evaluated the incidence of PE in wartime trauma patients early in the Iraq and Afghanistan conflicts. In their study, a disproportionate number of patients sustained fractures and/or amputations and they reported an incidence of PE of 5.7%, which is higher than the incidence commonly quoted for civilian trauma. They further noted that while multiple long bone fractures and pelvic fractures were independent risk factors of PE, VTE was more common in patients with amputations vs long bone fracture, suggesting a greater level of coagulopathy and/or vascular trauma with more severe extremity trauma. In our analysis, both PE and major VTE were more common than reported previously in this population. Univariate analysis demonstrated well-accepted injury constellations previously associated with VTE, including a greater severity of initial injury (i.e., higher ISS, lower GCS, greater base deficit, larger volume of blood products), were associated with major VTE in our population, potentially increasing this rate. Fractures of the vertebral bodies, pelvis, upper extremity, and lower extremity were each significantly associated with major thromboembolic events on univariate analysis, while multivariate analysis for injury characteristics specifically implicated trauma-related amputation and vertebral fracture. Several other injury and initial treatment characteristics were closely associated with the outcome on univariate analysis, including hand fractures and FFP administration, and were considered in multivariate analysis as predictor variables; however, these were not identified as significantly correlated with major VTE on regression models.

Over 70 randomized controlled trials evaluated the VTE risk of TXA during routine Orthopaedic surgeries and found no increased risk with its administration.^[29]^ Similarly, route of administration between intravenous and intrawound does not appear to affect rates of DVT or PE.^[30]^ Based on pooled data, the nontreatment cohorts surprisingly appear to be slightly higher risk for PE though this does not reach statistical significance.^[29]^ Conversely, some studies investigating TXA use in major civilian and military trauma have identified its use as an independent risk factor for VTE events. Myers et al^[31]^ performed a propensity score matching multivariate analysis of a large database of trauma patients and found nearly three-fold increased risk of VTE in patients receiving TXA. Adair et al^[32]^ observed a slightly increased risk of VTE in military trauma patients after TXA using LASSO regression, though patients were only included if they met criteria with ISS > 10 and received a massive transfusion. These study findings suggest that appropriate use in patients who receive TXA for massive hemorrhage likely has minimal effects on VTE risk, but effects are more problematic for the group of patients who do not reach this threshold. While TXA has been safely used in scheduled orthopaedic surgery for decades, the risk of VTE following TXA administration may be elevated following trauma due to the increased prothrombotic state.^[2,4,33]^ One suggested cause of trauma-related thrombotic events is acute coagulopathy of trauma, which is suspected to be due to the activation of protein C and concurrent inactivation of factors V and VIII.^[34]^ With high-energy trauma, fibrinolysis is dysregulated, and clotting or more lethal hyperfibrinolytic hemorrhage may occur.^[35]^ TXA, a lysine analog that binds to the lysine-binding sites on plasminogen, inhibits fibrinolysis, and stabilizes clots while decreasing their breakdown.^[9,34,36]^

While the mechanism for a potential increase in prothrombotic events is well defined, their clinical significance is still poorly defined. Nishihara and Hamada^[37]^ observed elevated prevalence of VTE in patients following total hip arthroplasty with intraoperative TXA than in those who did not receive routine DVT prophylaxis; however, they also noted that many of these VTEs were in the distal veins. Though these are counted as VTE events in their study, these events pose less clinical risk and do not typically necessitate treatment if they do not extend into proximal veins because they have a smaller likelihood of migrating to the lungs. Multiple authors have observed de novo PE formation in high-energy trauma with limited association to peripheral DVT, suggesting that while risk factors for these events are similar, they are not necessarily pathologically contiguous processes.^[17,21,24,38]^ We similarly observed no significant association between major VTE and DVT or a prior history of DVT.

There are several notable limitations to the current study. First, it is a retrospective, observational study, which is limited by certain biases including reliance on accuracy and completeness of the medical record. In light of these features of the study, there is a chance that false positive or negative predictor variables may have been included or excluded, respectively, for the multivariate model, resulting in a suboptimal model. We attempted to account for this with multiple imputations for missing variables; however, certain features that are truly associated with major VTE may not have occurred with sufficient frequency in our patient group to be detected. Another limitation was potential variability between practice patterns within providers throughout levels of care, such as the threshold to use TXA, fixation methods for fractures, and decision to complete early amputation versus attempt limb salvage. All patients within our study cohort met Tactical Combat Casualty Care guidelines for TXA administration based on anticipated need for massive transfusion, suggesting that low practitioner threshold to use TXA could account for increased risk.^[5]^ Last, it is important to recognize the generalizability and application of our data may be limited due to our study population. Our mean ISS was 21.5, indicating the average patient sustained major trauma, which variably correlates with increased risk of morbidity and mortality, including increased risk of VTE.^[39]^ Additionally, our population was almost entirely male, and given the overall low frequency of major VTE, the effects of sex could not be assigned a stable estimate.

Our models demonstrated an injury constellation including ISS > 23, major amputation, and vertebral fracture or a clinical constellation including TXA administration, traumatic amputation, acute kidney failure, hypertension, sedation at transfer, and greater time on ventilator are reliable for the prediction of major VTE after severe combat-trauma; however, it still requires validation in a larger patient cohort. Our models also determined that TXA administration, traumatic amputation, acute kidney failure, and hypertension were the most predictive of major VTE in both models controlling, and not controlling, for ISS. Factors identified by our clinical and injury models are similar to those seen in separate studies of risk factors for VTE after severe military and civilian trauma.^[10,23,26,40,41]^ Similar predictive models have also implicated several risk factors we identified including injury severity, spinal injury, and other orthopaedic injuries, with a similar predictive power, although these did not evaluate TXA use and none exclusively considered patients with orthopaedic injuries.^[42–44]^

If VTE events are increased in frequency in patients who receive TXA after major military trauma, it is imperative to determine the clinical relevance of these events as the potentially life-saving benefits of TXA largely outweigh these risks, particularly for DVT and subsegmental PE. In our study, TXA was an independent predictor of major, life-threatening VTE event, which suggests it is worthwhile to further refine our use of this agent and/or adjust management for patients with risk factors identified in this study who do receive TXA. To date, this is the only study that we are aware of looking at the risk of major thromboembolic events following TXA administration with a focus on orthopaedic injuries rather than hemorrhage from another source, particularly in a military population. Our analysis suggests that orthopaedic injuries increase the incidence of major thromboembolic events following combat-related trauma compared with the general trauma populations. In particular, injuries including traumatic amputations and vertebral fractures are associated with greatest risk and should prompt careful attention to development of thromboembolism. However, additional prospective investigation will be necessary to calibrate patient selection for TXA administration and risk stratification for patients who do receive TXA to assist providers in optimizing protective factors against development of major thromboembolic events.
